# Physical function patient‐reported outcomes among adolescent and young adult cancer survivors: A systematic review

**DOI:** 10.1002/cam4.7046

**Published:** 2024-03-23

**Authors:** Sarah Tanner, Teyl Engstrom, Cheryl Forbes, Dhaval Patel, Wen Ray Lee, Rick Walker, Natalie Bradford, Jason D. Pole

**Affiliations:** ^1^ Centre for Health Services Research The University of Queensland Herston Queensland Australia; ^2^ Child Health Research Centre The University of Queensland South Brisbane Queensland Australia; ^3^ School of Medicine The University of Queensland Herston Queensland Australia; ^4^ Queensland Children's Hospital Brisbane Queensland Australia; ^5^ Princess Alexandra Hospital Brisbane Queensland Australia; ^6^ Cancer and Palliative Care Outcomes Centre at Centre for Children's Health Research Queensland University of Technology Brisbane Queensland Australia; ^7^ The University of Toronto Dalla Lana School of Public Health Toronto Ontario Canada

**Keywords:** adolescent, cancer, cancer survivors, patient‐reported outcome measures, physical function, routinely collected health data, young adult

## Abstract

**Background:**

The physical challenges faced by adolescents and young adults (AYA) after a cancer diagnosis may be different from those experienced by paediatric and older adult cancer patients. Patient‐reported outcome measures (PROMs) are valuable tools that can be useful in exploring the experiences of AYAs and identifying important issues, recurrent themes and areas to potentially improve quality of life.

**Objective:**

We compared patient‐reported physical function outcomes between AYAs diagnosed with cancer and non‐cancer controls.

**Method:**

This paper builds on a scoping review published in early 2023 and focuses on PROMs related to physical function.

**Results:**

This systematic review includes 16 studies that measured and reported on physical function PROMs in AYA cancer survivors compared with their cancer‐free peers. Of these studies, 14 found that physical function in AYA survivors was significantly worse. This paper also includes a meta‐analysis conducted on 5 studies using the EORTC‐QLQ‐C30 to measure physical function, which found that physical function score was an average of 7.03 (95% CI: −10.21, −3.86) points lower in the AYA cancer group, compared to their cancer free‐peers, a difference that is clinically meaningful.

**Conclusions:**

The results overwhelmingly demonstrate that AYAs post a cancer diagnosis have worse health‐related quality of life from a physical function perspective than their cancer‐free peers, providing a compelling argument for the need to address this issue. All but one of the studies were cross‐sectional, which highlights the need for further assessment of this group longitudinally throughout their cancer journey.

## Graphical abstract

We identified 16 eligible studies, 14 (87.5%) of which found those diagnosed with cancer reported poorer physical functioning than the non‐cancer controls. A meta‐analysis of 5 studies which used the EORTC‐QLQ‐C30 to measure physical function found that the physical function score was an average of 7.03 (95% CI: −10.21, −3.86) points lower in the AYA cancer group, compared to the controls, which is a clinically meaningful difference.

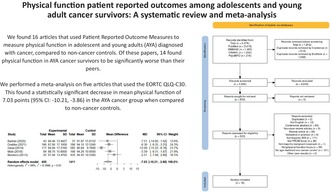

## 1 INTRODUCTION

In developed countries, survival rates are improving among adolescent and young adult (AYA) cancer patients, with the most recent 5‐year survival rate sitting at approximately 85%.^1^, ^2^, ^3^ As a result, the majority of AYAs with cancer survive for a long period of time after diagnosis and treatment.^4^ Given the prolonged survival, investigation is required to understand and address the impact of a cancer diagnosis on the quality of life of AYA cancer patients during the entire survivorship period including at the time of diagnosis and treatment, and throughout their time in remission.

Eastern Cooperative Oncology Group (ECOG) status is a measure of the daily functionality of a cancer survivor, and physical function is an important component of the scale.^5^ Research studies in general have identified poorer physical function outcomes in cancer patients and survivors compared to healthy populations. For example, myeloma patients across all age groups are reported to have worse aerobic capacity and muscle strength when compared to normative data.^6^ Notably, the myeloma group obtained mean results on a 6‐minute‐walk‐test that were less than the reference line for one standard deviation below the normative population mean.^6^ In a geriatric population, cancer patients had increased impairment of instrumental activity of daily living (IADL) and physical activity, as well as lower scores on the short physical performance battery (SPPB).^7^ A study of 79 women (40 years and over) undergoing breast cancer treatment found they had significantly lower functional status than the general population, as measured by handgrip strength, a 6‐minute walk test, and a bio‐impedance phase angle.^8^ Their function decreased as they underwent treatment, with chemotherapy profoundly affecting functionality.^8^ A focus on AYAs, defined as people aged 15–39 years,^1^ is crucial because most research into cancer survivors has been centred around children or older adult patients.^9^

While anthropometric data are an important component in assessing physical function, a patient's subjective experience of their condition and functioning is also key in determining their quality of life. Patient‐reported outcome measures (PROMs) have been used to evaluate the impact of cancer on AYAs. PROMs are instruments or tools that are used to collect information directly from patients in the form of questionnaires.^10^ This allows AYA cancer patients to voice their experiences meaningfully, bringing attention to information that could otherwise be missed by their clinical team. The information is collected through questionnaires or surveys that are often standardised and validated, which allows assessments to be made over time and between different groups, including comparison with peers who have not been confronted with a cancer diagnosis.

This study builds on the work of a systematic review previously published that identified the areas investigated by PROM tools used in AYA cancer patients to understand patient‐reported challenges and outcomes.^11^ In this specific study, we aimed to answer the question: how do physical function outcomes, as measured by PROM tools, differ between AYA cancer survivors and their cancer‐free peers?

## 2 METHODS

The initial systematic review is described fully elsewhere.^11^ Briefly, four databases (PubMed, EMBASE, CINAHL and PsycINFO) were searched for manuscripts published between 1 January 2011 and 16 June 2021. We aimed to find manuscripts that focused on AYAs (ages 15–39) who had been diagnosed with malignant neoplasms and included measurement using a PROM tool. The search strategy used included subject headings and related free text searches. An example of the search terms from our PubMed search is as follows: “(teen* OR adolesce* OR "adolescent"[MeSH Terms] OR "young adult" OR "Young Adult"[Mesh]) AND ("Neoplasms"[Mesh] OR Neoplasms OR cancer[Title/Abstract] OR malignancy OR chemo*) AND ("Patient Reported Outcome Measures"[Majr] OR "Patient Outcome Assessment"[Majr] OR "Quality of Life"[Majr])”. The strategy was also developed with a professional librarian. The methods used in this study are similar to another systematic review, which this group has published, which focuses on AYA cancer patient‐reported outcomes in the mental health domain.^12^

We used the following inclusion criteria: AYA‐focused (either two‐thirds of study cohort aged 15–39 years at diagnosis, or results for subjects in this age group reported separately); diagnosed with a malignant neoplasm; and PROM as a central outcome. We excluded manuscripts that reported only on PROM protocols, validation studies or outcomes for family or caregivers.

Manuscripts initially underwent a title and abstract review, followed by full‐text review included additional exclusion criteria: inability to identify full text; not written in English; or review article. Both steps were conducted independently by two out of three reviewers (TE, ST and WRL). Discussion at a team meeting with all three authors was used to obtain consensus following each step in case of disagreement. For title and abstract review, Cohen's Kappa was 0.59, representing moderate agreement. For the full‐text review, Cohen's Kappa was 0.69, representing substantial agreement. Finally, we undertook data extraction for the relevant information from the included papers.

For this review, manuscripts identified that measured physical function were considered for inclusion and re‐reviewed to filter to only papers which included a cancer‐free AYA comparison group. We conducted a reverse reference search of all identified manuscripts to identify additional relevant studies that may have been missed in the initial search and review. Finally, the included manuscripts underwent additional data extraction, in which details of the physical function measures along with the outcomes for both AYAs with and without cancer was obtained. Physical function was taken to include measures of ‘physical functioning’, ‘physical function’ and ‘physical component summary’.

Two authors assessed each study using the Risk of Bias In Non‐randomised Studies of Interventions (ROBINS‐I) tool.^13^ The studies were assessed independently by each author, and a meeting was then held during which any discrepancies were discussed and resolved. Risk of bias was assessed in the following seven domains: confounding; selection of participants; classification of intervention; deviations from intended interventions; missing data; measurement of outcomes; and selection of reported results.^13^ Each domain had four categories for risk level: low; moderate; serious; or critical.^13^ Each domain received an individual rating, and the overall risk of bias rating given to the study was the least favourable score in any domain.^13^

A meta‐analysis was performed on studies that used the most prevalent measure of physical function—the European Organisation for Research and Treatment of Cancer—Quality of Life Questionnaire (EORTC‐QLQ‐C30).^14^ One study that used this tool was not included because the required data were not available in the article. The meta‐analysis used the R statistical software package ‘meta’ using a random effects model.^15^

## 3 RESULTS

### 3.1 Study characteristics

Eighteen articles met the initial criteria, but 2 were removed after further evaluation because they did not meet subsequent criteria. One study met the control group criteria but did not compare the results of the physical function PROM against the control group.^17^ One measured physical function as part of a general health‐related quality of life score but only reported the overall score. It did not measure physical function in the control group to provide a comparison.^18^ In the reverse reference search, no further articles were identified that matched the inclusion criteria.

**FIGURE 1:**
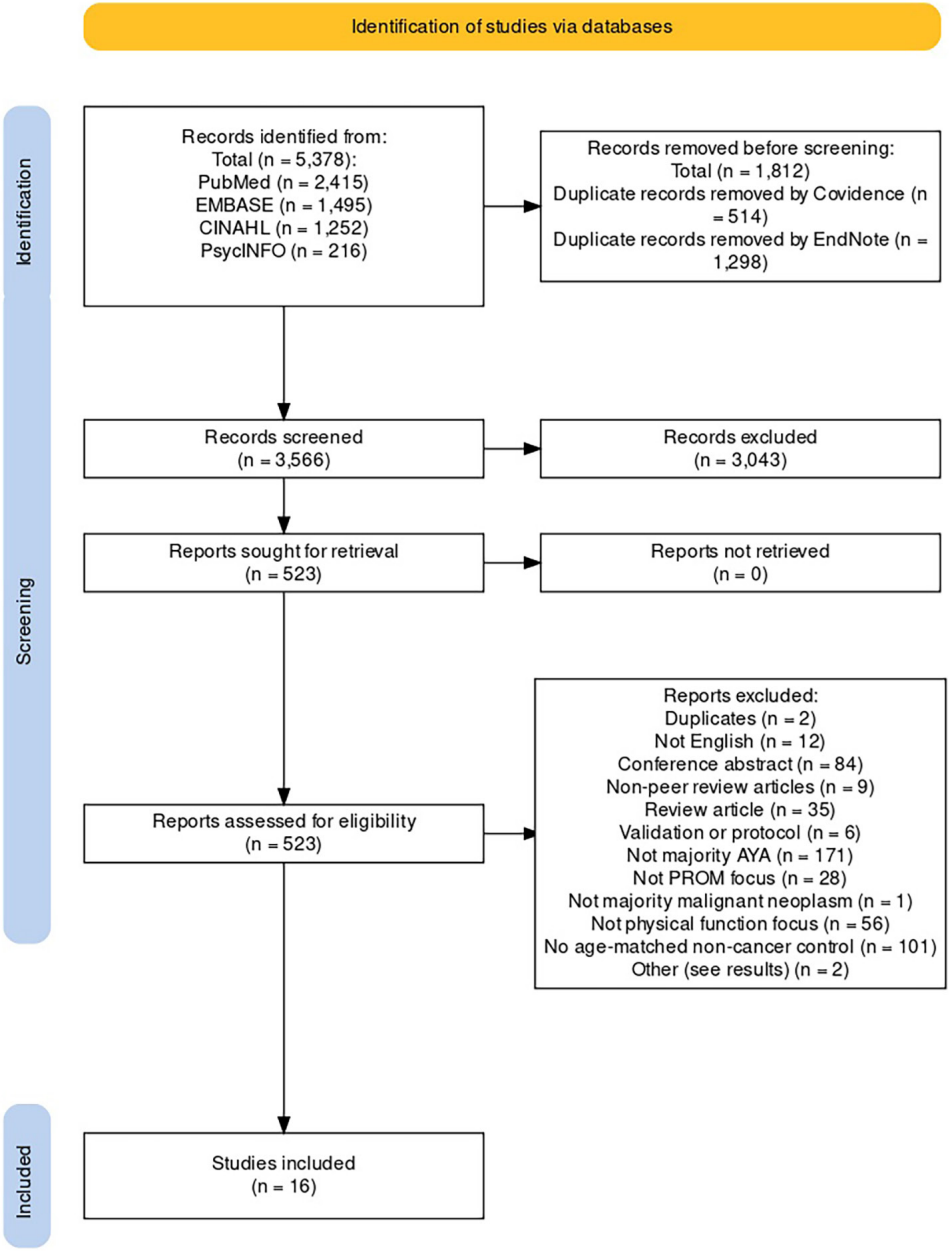
PRISMA study selection diagram.^16^ From: Page MJ, McKenzie JE, Bossuyt PM, Boutron I, Hoffmann TC, Mulrow CD, et al. The PRISMA 2020 statement: an updated guideline for reporting systematic reviews. BMJ 2021;372:n71. doi: 10.1136/bmj.n71. For more information, visit: http://www.prisma‐statement.org/.

Therefore, 16 articles were included in the final review (Figure 1). Table 1 contains the study characteristics and Table 2 contains the study details. Fourteen studies were cross‐sectional, while 1 was a cohort study. The final study consisted of a cohort study of one AYA cancer group and a cross‐sectional study of a second cancer group as well as the control group. Eight of the 16 were published in 2015 or later; the other 8 were published between 2011 and 2014. Eight of the studies were conducted in Europe. One was conducted in Australia, and the other 7 were North American studies—6 from the USA and 1 from Canada.

**TABLE 1:** Characteristics of included studies.

Characteristic		Number (%)
Study type	Cross‐sectional	14 (87.5)
	Cohort	1 (6.25)
	Cross‐sectional cohort	1 (6.25)
Geographical area	North America	7 (43.75)
	Europe	8 (50)
	Australia	1 (6.25)
Year of publication	2015 onwards	8 (50)
	2011–2014	8 (50)
Scale used	EORTC‐QLQ‐C30	6 (37.5)
	SF‐12	3 (18.75)
	BRFSS	3 (18.75)
	FACT‐G	2 (12.5)
	SF‐36	1 (6.25)
	FLZ	1 (6.25)
Inclusion criteria	All cancers	10 (62.6)
	Limited to certain cancers	2 (12.5)
	Breast cancer	1 (6.25)
	Thyroid cancer	1 (6.25)
	Sarcomas	1 (6.25)
	Cervical cancer	1 (6.25)

**TABLE 2:** Summary of included studies.

Study	Study Design	Country	Patient characteristics	Disease type	Comparison group	Survey used	Results
Bartolo 2020^19^ ‘Fertility under uncertainty: exploring differences in fertility‐related concerns and psychosocial aspects between breast cancer survivors and non‐cancer infertile women’	Cross‐sectional	Portugal	Breast cancer survivors *N* = 43 Mean age (at enrolment) = 36.16 (3.11) years; range = 26–40 Mean age (at diagnosis) = 33.40 (3.81) years	Breast cancer	Healthy controls *N* = 37 Mean age (at enrolment) = 32.41 (3.87) years; range = 25–40	EORTC	Physical functioning—breast cancer survivors: 84.46; non‐cancer infertile women: 92.24; controls: 91.97. Cancer survivors had significantly worse physical functioning than non‐cancer infertile women but not than controls.
Bradford 2020^20^ ‘Do specialist youth cancer services meet the physical, psychological, and social needs of adolescents and young adults? A cross sectional study’	Cross‐sectional	Australia	*N* = 42 Male = 18 (43%) Female = 24 (57%) Age at diagnosis: 10–14 years *n* = 5 (12%) 15–19 year *n* = 17 (40%) 20–24 years *n* = 16 (38%) 25–29 years *n* = 4 (10%) Age at study: 15–19 years *n* = 13 (31%) 20–24 years *n* = 18 (43%) 25–29 years *n* = 8 (19%) 30+ *n* = 3 (7%)	Any form of invasive cancer Leukaemia or lymphoma *n* = 20 (48%) Brain cancer *n* = 8 (19%) Bone or soft tissue sarcoma *n* = 9 (21%) Other *n* = 5 (12%)	Queensland population norms for 20‐ to 39‐year‐olds	FACT‐G	Quality of life was significantly lower in AYAs compared to population norms across physical, social, emotional and functional well‐being, with physical and emotional well‐being accounting for the most variation.
Drabbe 2021^21^ ‘The age‐related impact of surviving sarcoma on health‐related quality of life: data from the SURVSARC study’	Cross‐sectional	Denmark	*N* = 186 Male = 84 (45.2%) Female = 102 (54.8%) Median age at diagnosis = 30 Median age at questionnaire = 37	Sarcomas‐Excluded: desmoid fibromatosis, Grade I chondrosarcoma, gastrointestinal stromal tumours, atypical lipomatous tumours, giant cell tumours	Age‐matched and sex‐matched normative sample	EORTC‐QLQ‐C30	Statistically significant worse physical function in AYA survivors compared with normative population.
Froding 2014^22^ ‘Quality of life, urogynecological morbidity, and lymphedema after radical vaginal trachelectomy for early‐stage cervical cancer’	Cross‐sectional cohort	Denmark	RVT group *N* = 18 Median age – 29 (23–42) RAH group *N* = 32 Median age – 42 (30–63)	Cervical cancer stage IA2 to IB1	Nurses and students from the department of gynaecology *N* = 30 Median age – 28.5 (24–41)	EORTC‐QLQ‐C30	RVT group were not statistically significantly different from (lower than) controls at baseline but were at 3‐, 6‐ and 12‐month marks. RAH group was statistically significantly different from (lower than) controls at their measurement point of 12 months.
Geue 2013^23^ ‘Gender‐specific quality of life after cancer in young adulthood: a comparison with the general population’	Cross‐sectional	Germany	*N* = 117 Women = 65.81% Men = 34.19% Mean age at survey = 31.3 (5.96) Mean time since diagnosis = 29 months	Any cancer	Two representative German survey samples from 1998 and 2012 *N* = 585 Men *n* = 200 Women *n* = 385 Mean age = 31.17 (5.86)	EORTC‐QLQ‐C30	Lower mean physical function score in AYA than representative sample. There was an interaction with gender, suggesting that physical function in female cancer patients have poorer quality of life in terms of physical function
Harju 2018^24^ ‘Health‐related quality of life in adolescent and young adult cancer survivors’	Cross‐sectional	Switzerland	*N* = 155 Female = 59 (38.1%); Male = 96 (61.9%) Age at diagnosis = 16–20 *n* = 67 21–25 *n* = 88 Mean = 21.6 (2.89) Mean age at study = 34 (5.87)	CNS tumours, germ cell tumours, lymphomas, leukaemias, neuroblastomas, renal, hepatic and bone tumours, as well as soft tissue sarcomas. Lymphomas (38%) and germ cell tumours (29%) most common.	Random sample of Swiss general population. Age group 20–47 years included. *N* = 350; Males = 140 (40%); Mean age = 35.5 years	SF‐12	Survivors with a migration background, who were unemployed, or had late effects, were more likely to report poor physical health, as did those who had compulsory education only (in univariable regression). Only migration background and unemployment were significantly associated with poor physical health.
Husson 2017^25^ ‘Health‐Related Quality of Life in Adolescent and Young Adult Patients with Cancer: A Longitudinal Study’	Cohort	USA	Cancer patients *N* = 176 at T1; *n* = 141 at 24‐mo follow‐up Mean age at diagnosis = 23.6 (8.9) years Male = 55.1% (97); Female = 44.9% (79) Age at diagnosis: 14–17 years *n* = 80 (45.5%) 18–25 years *n* = 35 (19.9%) 26–39 *n* = 61 (34.7%) On treatment *n* = 166 (94.3%) Off treatment *n* = 10 (5.7%)	Any invasive cancer	US population norms—weighted means and pooled standard deviation for the 18–44 years age group	SF‐36	Clinically relevant difference in AYA patients and population norms for physical functioning.
Kirchoff 2014^9^ ‘Sociodemographic Disparities in Quality of Life for Survivors of Adolescent and Young Adult Cancers in the Behavioral Risk Factor Surveillance System’	Cross‐sectional	USA	*N* = 8375 Current age: 20–29 age group: *n* = 350 30–39 age group: *n* = 1236 40–64 age group: *n* = 4935 65+ age group: *n* = 1854 Males = 242 (23.9%); Females = 1344 (76.1%) Age at diagnosis: 15–29 age group: *n* = 3638 30–39 age group: *n* = 3981	Any cancer	*N* = 334,759 Male = 50.2% (*n* = 24,901); Female = 49.8% (*n* = 40,671) 20–29 age group: *n* = 21,254 (44.6%) 30–30 age group: *n* = 44,318 (55.4%)	BRFSS	Number of poor physical function days per month for survivors exceeded controls in both men and women
Mols 2018 ‘Age‐related differences in health‐related quality of life among thyroid cancer survivors compared with a normative sample: results from the PROFILES registry’	Cross‐sectional	The Netherlands	Thyroid cancer survivors *N* = 84 Mean age at diagnosis = 29.1 (5) Mean time since diagnosis = 11 (5.4) years Time since diagnosis: <5 years *n* = 15 (17.9%) 5–10 years *n* = 20 (23.8%) >10 years *n* = 49 (58.3%) Male *n* = 15 (17.9%) Female *n* = 69 (82.1%)	Thyroid cancer	Normative sample obtained from 2009 wave of Health and Health Complaints project from CentERdata, involving a random sample of Dutch adults. Age‐matched and sex‐matched selected from this group.	EORTC‐QLQ‐C30	There was a statistically significant but small clinically important difference between the AYA survivors and normative population in physical functioning.
Monteiro 2013 (19) ‘Psychosocial Outcomes in Young Adults with Cancer: Emotional Distress, Quality of Life and Personal Growth’	Cross‐sectional	Portugal	*N* = 36 Age range: 20–38 Mean age 28.53 (5.13) 12 M (33.3%); 24 F (66.7%) 11 in treatment phase; 25 off‐treatment survivors. Cancer patients: age range 20–36; M = 28, SD 6.13; diagnosed average of 1.73 years (SD 1.19) before study Off‐treatment survivors: age range 22–38; M = 28.76, SD 4.75; diagnosed average of 3.96 years (SD 2.75) before study	Any cancer	*N* = 435 Age range: 24–39 Mean age 28.86 (4.12) 149 M (34.3%) 286 F (65.7%)	EORTC‐QLQ‐C30	No statistically significant difference in physical function between cancer patients and controls.
Salsman 2014^26^ ‘Physical, Emotional, and Social Health Differences Between Posttreatment Young Adults With Cancer and Matched Healthy Controls’	Cross‐sectional	USA	*N* = 335 Mean age 31.8 (SD = 5.4) Female *N* = 229 (68.4%) 0–12 months post‐treatment: *n* = 120; 35.8% 13–24 months post‐treatment: *n* = 102; 30.4% 25–60 months post‐treatment: *n* = 113 33.7%	Any cancer	*N* = 335 Mean age 31.8 (SD = 5.4) Female *N* = 229 (68.4%)	FACT‐G	Worse physical well‐being scores in AYA group compared with the healthy control group, with a > 3 point difference in scores, indicating the difference is meaningful.
Schulte 2021^4^ ‘Quality of Life Among Survivors of Adolescent and Young Adult Cancer in Canada: A Young Adults With Cancer in Their Prime (YACPRIME) Study’	Cross‐sectional	Canada	AYA Cancer Survivors *N* = 195 Male: *N* = 36 (17.8%) Female: *N* = 166 (82.2%) >2 years post‐therapy completion Age at diagnosis: 27.74 (6.27) Age at time of study: 20–29 *n* = 31 (15.4%) 30–39 *n* = 116 (57.4%) 40–49 *n* = 50 (24.8%) 50–64 *n* = 5 (2.5)	Any cancer	Male: *N* = 141 (21.2%) Female: *N* = 524 (78.8%) Age at time of study: 20–29 *n* = 147 (22.1%) 30–39 *n* = 333 (50.1%) 40–49 *n* = 58 (23.8%) 50–64 *n* = 27 (4.1%)	SF‐12 (AYA survivor) vs. SF‐36 (CCHS)	Significantly lower physical health in survivors compared with comparison sample.
Seitz 2011^27^ ‘Life satisfaction in adult survivors of cancer during adolescence: what contributes to the latter satisfaction with life?’	Cross‐sectional	Germany	*N* = 820 Mean age 30.4 (6.1) Female = 418 (51%) Male = 402 (49%)	Any cancer	Controls general Life Satisfaction *N* = 1946 Mean age = 30.44 (6.05) Males = 954 (49%) Females = 992 (51%) Controls health‐related Life Satisfaction *N* = 633 Mean age = 30.44 (6.05) Males = 310 (49%) Females = 323 (51%)	Questions on Life Satisfaction FLZ	No statistically significant difference in life satisfaction from a mobility perspective between survivors and community samples
Smith 2013^28^ ‘Health‐Related Quality of Life of Adolescent and Young Adult Patients with Cancer in the United States: The Adolescent and Young Adult Health Outcomes and Patient Experience Study’	Cross‐sectional	USA	AYA Cancer patients 6‐ to 14‐months post‐diagnosis *N* = 523 Male: *N* = 331 (63.3%) Female: *N* = 192 (36.7%)	Non‐Hodgkin lymphoma, Hodgkin lymphoma, germ cell cancer, acute lymphocytic leukaemia, or sarcoma	US population norms—age‐matched	SF‐12 and PedsQL	Worse physical functioning than in the reference population, statistically significantly.
Tai 2012^29^ ‘Health Status of Adolescent and Young Adult Cancer Survivors’	Cross‐sectional	USA	*N* = 4054 AYA cancer diagnosed at ages 15–29 years M = 18.9% F = 81.1% Median age = 40.2	Any cancer	*N* = 345,592 M = 49.2%; F = 50.8%	BRFSS	More AYA cancer survivors reported no leisure‐time physical activity in the past month compared with respondents who had no history of cancer. AYA cancer survivors also more likely to report at least 14 days of poor physical health in the past month compared with controls.
Warner 2016^30^ ‘Health behaviors, quality of life, and psychosocial health among survivors of adolescent and young adult cancers’	Cross‐sectional	USA	*N* = 7619 Current age [Females]: 20–29 *n* = 152 (5.9%) 30–39 *n* = 693 (17.5%) 40–64 *n* = 4028 (60.8%) >/=65 *n* = 1568 (15.8%) Age at diagnosis [Females] 15–20 *n* = 665 (15.4%) 20–29 *n* = 2516 (38.8%) 30–39 *n* = 3260 (45.8%) Current age [Males]: 20–29 *n* = 17 (3.8%) 30–39 *n* = 106 (16.9%) 40–64 *n* = 769 (66.7%) >/=65 *n* = 286 (21.6%) Age at diagnosis [Males] 15–20 *n* = 136 (14.5%) 21–29 *n* = 321 (29.1%) 30–39 *n* = 721 (56.4%)	Any cancer	*N* = 334,759 Current age [Females]: 20–29 *n* = 12,969 (16.6%) 30–39 *n* = 27,702 (21.2%) 40–64 *n* = 105,356 (45.4%) >/=65 *n* = 61,064 (16.9%) Current age [Males]: 20–29 *n* = 8285 (18%) 30–39 *n* = 16,616 (21.8%) 40–64 *n* = 71,307 (48%) >/=65 *n* = 31,460 (12.2%)	BRFSS	Both female and male survivors had more days of poor physical health than male controls.

Six studies used the EORTC‐QLQ‐C30 to assess physical function. Three used the Short Form 12 (SF‐12). Another three studies collected information via the nationwide health survey the 2009 Behavioral Risk Surveillance System (BRFSS) survey instead of a specific PROM tool. Two studies used the Functional Assessment of Cancer Therapy (FACT‐G) questionnaire, one used the Questions of Life Satisfaction (FLZ) and one used the Short Form 36 (SF‐36).

Ten of the papers included all forms of cancer. Two studies limited participants to a list of specific cancers (CNS tumours, germ cell tumours, lymphomas, leukaemias, neuroblastomas, renal, hepatic and bone tumours, as well as soft tissue sarcomas) so that comparison with outcomes for childhood cancer patients was possible. One looked at breast cancer, one examined thyroid cancer, one cervical cancer and one sarcoma.

The risk of bias assessment of the included studies is presented in Table 3. Eleven studies were deemed moderate risk of bias and five studies low risk. Eight (50%) of the papers scored moderate risk for bias in the category of selection of participants into the study. Four (25%) scored moderate risk for bias in the category of confounding, three (19%) scored moderate risk in the category of bias due to measurement of outcomes and one (6%) scored moderate risk in the category of bias due to missing data.

**TABLE 3:** Risk of bias assessment of included studies.

Study		Pre‐intervention		At intervention	Post‐intervention				Overall risk of bias
First author	Year	Bias due to confounding	Bias due to selection of participants into the study	Bias in classification of interventions	Bias due to deviations from intended interventions	Bias due to missing data	Bias in measurement of outcomes	Bias in selection of the reported result	Severity
Smith	2013	Low	Moderate	Low	Low	Low	Low	Low	Moderate
Drabbe	2021	Low	Moderate	Low	Low	Low	Low	Low	Moderate
Froding	2014	Low	Moderate	Low	Low	Moderate	Moderate	Low	Moderate
Mols	2018	Low	Moderate	Low	Low	Low	Low	Low	Moderate
Seitz	2011	Low	Moderate	Low	Low	Low	Low	Low	Moderate
Geue	2014	Low	Low	Low	Low	Low	Low	Low	Low
Bradford	2020	Low	Moderate	Low	Low	Low	Low	Low	Moderate
Bartolo	2020	Low	Low	Low	Low	Low	Low	Low	Low
Kirchoff	2014	Moderate	Low	Low	Low	Low	Low	Low	Moderate
Monteiro	2013	Moderate	Moderate	Low	Low	Low	Low	Low	Moderate
Salsman	2014	Low	Low	Low	Low	Low	Low	Low	Low
Schulte	2021	Low	Moderate	Low	Low	Low	Moderate	Low	Moderate
Tai	2012	Moderate	Low	Low	Low	Low	Low	Low	Moderate
Warner	2016	Low	Low	Low	Low	Low	Low	Low	Low
Harju	2018	Low	Low	Low	Low	Low	Low	Low	Low
Husson	2017	Moderate	Low	Low	Low	Low	Moderate	Low	Moderate

### 3.2 Participant characteristics

The three BRFSS papers studied 8375, 7619 and 4054 participants and all included all types of cancer, except non‐melanomatous skin cancers. Each of these studies selected participants from the same primary data source, and therefore we anticipate a marked overlap of subjects across these three papers. The exact degree of overlap is difficult to assess since each study had slightly different exclusion and inclusion criteria in terms of the extent of the US territories included or the time from cancer diagnosis. Due to the overlap, we report patient characteristics from the 10 non‐BRFSS studies, which included a total of 2762 cancer participants. These participants were AYA cancer patients at the time of the study but also longer‐term survivors who had a diagnosis of cancer in their AYA years.

Four studies reported mean age at time of study; three studies reported mean age at diagnosis; and a further two studies reported both. Two studies reported median age at study, while one study reported both median age at study and median age at diagnosis. Four studies reported no measure of central tendency to describe age at either study or diagnosis, but instead categorised the number of patients into specific age brackets. Of those studies that did not report mean age at diagnosis, one group was recently post‐operative; in another study the patients were on average 29 months from diagnosis; and another two gave a range of 0–60 months and 6–14 months post diagnosis.

Of the papers providing mean age at study, participants were between the ages of 28 and 37. In the papers providing mean age at diagnosis, the range was from 21 years of age to just under 34 years of age at diagnosis.

All studies reported on the sex of participants. There was a total of 1532 (55.3%) women and 1237 (44.7%) men. One study contained a discrepancy of 7 participants between the number reported overall, and the number of men and women participating in the study (reporting that *n* = 195, with 36 men and 166 women), which explains the small discrepancy between the totals.

### 3.3 Physical function outcomes

Fourteen studies (87.5%) reported that AYAs reported statistically significantly worse outcomes on PROM tools measuring physical function. One study, which used the EORTC‐QLQ‐C30, found no statistically significant difference between the two groups.^31^ Another study found no statistically significant difference in life satisfaction from the perspective of mobility between the two groups.^27^ None of the studies reported further analysis of the risk factors in this group for poorer physical function outcomes.

With regards to the 14 studies that identified statistically significantly worse physical function outcomes, the most used PROM tool was the EORTC‐QLQ‐C30 which was used by five studies.^19^, ^21^, ^22^, ^23^, ^32^ Three of those studies reported differences in physical function score between 5 and 10^19^, ^21^, ^32^; one study reported a larger difference of 12.^23^ The fifth found that the cancer group scored 10 points lower at 3 and 6 months than the control group baselines on physical function, though only the 3‐month interval was statistically significant.^22^ For the EORTC‐QLQ‐C30 the minimal important difference is generally considered to be 5–10.^33^, ^34^ Three studies used the SF‐12 Physical Component Summary and reported differences between the AYA cancer patients and healthy controls of 2.2,^24^ 3.0^28^ and 9.2^4^ points. Two studies used the FACT‐G, reported the AYA cancer patients scored lower than healthy controls by 1.5^26^ and 5.0 points.^20^ Three studies used the BRFSS and reported on the percentage who reported at least 15 days of poor physical health per month, which was 11.5,^9^ 12.2^30^ and 13.8 percentage points^29^ higher in the AYA cancer group compared to healthy controls. The remaining study used the SF‐36 physical function domain and reported AYA cancer patients scored on average 32.9 points lower at baseline than the US normative population, though this decreased to a difference of 13.4 by 24 months later.^25^

### 3.4 Physical function associations

Few studies looked at associations between worse physical function outcomes with patient demographics. One study found that female cancer patients have worse self‐reported physical functioning compared to their male counterparts, though worse physical function outcomes among women were also seen in the non‐cancer sample.^23^ Another study by Harju et al. (2018) identified that patients who had a migration background (OR = 5.34, CI 1.87–15.26), who were experiencing late effects of treatment (OR = 2.75, CI 1.00–7.67), who were unemployed (OR = 8.86, CI 2.61–30.16) or had only been educated to the compulsory level (OR = 2.37, CI 0.54–10.42), were more likely to report poor physical health in univariable regression.^24^ The only factors that were significantly associated with poor physical health in multivariable regression, however, were having a migration background (OR = 4.63, CI 1.50–14.28) and being unemployed (OR = 7.66, CI 1.93–30.34).^24^

### 3.5 Meta‐analysis

**FIGURE 2:**
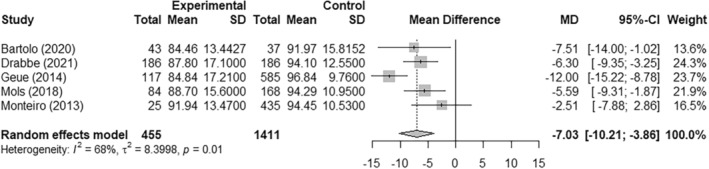
Meta‐analysis results.

A meta‐analysis was conducted on the studies by Bartolo,^19^ Drabbe,^21^ Geue,^23^ Monteiro^31^ and Mols^32^ using ‘R package meta’ and a random effects model. These studies measured physical function using the European Organisation for Research and Treatment of Cancer – Quality of Life Questionnaire (EORTC‐QLQ‐30) PROM tool.^14^ Note that only the cancer survivors were included from the Monteiro study, not those currently on treatment, for consistency with the other studies. While Froding^22^ also used EORTC‐QLQ‐C30, standard deviations were not presented in the paper and the study used a different study design, and hence was excluded from the meta‐analysis. The meta‐analysis consisted of 466 patients and 1411 controls. All five studies individually reported poorer physical function in the AYAs with cancer compared to non‐cancer peers, four of which were statistically significant results. The mean difference in physical function scores between the groups was −7.03 (95% CI: −10.21, −3.86). This shows a statistically significant mean decrease in physical function of 7.03 points (scale 0–100) in the cancer survivor group when compared to the non‐cancer control group. For the EORTC‐QLQ‐C30 the minimal important difference is generally considered to be 5–10.^33^, ^34^ Two of the studies included in the meta‐analysis were classified as moderate risk of bias,^21^, ^31^ while three were low risk of bias.^19^, ^23^, ^32^ Results of the meta‐analysis can be found in Figure 2.

## 4 DISCUSSION

In this systematic review of 16 studies, we described the patient‐reported physical function outcomes in AYAs with cancer, compared with AYAs without cancer. Most (14 out of 16, 87.5%) studies found that physical function outcomes among AYA cancer patients and survivors were statistically significantly worse than among their cancer‐free peers. Our meta‐analysis of five studies, which used the EORTC‐QLQ‐30 PROM tool, further illustrated this, finding AYAs with cancer reported physical function and average of seven points below those without cancer; a result, which exceeds the minimally important difference threshold of 5 points.^33^, ^34^

Cancer patients and survivors can experience many forms of physical function impairment due to their treatment, comorbidities or both.^35^ Examples can include pain, peripheral neuropathy, or insomnia, as well as both acute and long‐lasting toxicities resulting from treatment.^35^ There is scope for further work into the specific complaints experienced by AYA cancer patients and survivors leading to their poorer self‐reported physical function, so that efforts can be made to reduce this difference and improve their overall quality of life.

In addition to the clear statistically significantly lower physical functioning among the cancer group compared to healthy controls, most studies also revealed clinically meaningful differences. Of the six overall studies using EORTC‐QLQ‐C30, three reported results within 5–10 points difference in the range of the minimally meaningful difference, while one study showed a moderate change with a difference of 12.0.^33^, ^34^ For the SF‐12 PCS, the studies reported differences of 2.2, 3.0 and 9.2; a systematic review of PROMs suggests the minimally important difference for this measure is 4–7.^36^ For studies using FACT‐G, differences of 1.5 and 5 were observed, only one of which is within the clinically meaningful difference range of 5–6 points.^36^ Husson used the SF‐36 and reported cancer patients scored 14.1 below the population norm group, which represents a large clinically meaningful difference.^37^ The remaining studies reported the percentage who reported at least 15 days of poor physical health per month, with results between 11.5 and 13.8 percentage points, rather than using a point‐based scoring system. It should be noted that the definitions of clinically meaningful differences vary by cancer type, and by cohort and much of the research is not specific to AYAs with cancer; research in this area is ongoing.^33^, ^38^

Though most studies were consistent in the physical function outcomes, surprisingly few studies explored factors associated with worse physical function in more detail. Those who were female,^23^ from a migration background or who were unemployed had poorer physical function scores.^24^ The absence of further detailed analysis by most papers of factors associated with poorer physical function is likely due to the papers examining multiple PROM outcomes at one time, as opposed to focusing on physical function. This is therefore a potential area for future investigation, to identify those sub‐populations that are most at risk of poor physical functioning and develop interventions to address the specific physical functioning challenges among those most impacted.

The worse self‐reported physical function among AYA cancer patients and survivors, almost across the board, is in line with previous research. One population‐based study found that physical performance limitations were found in over half of cancer survivors, compared with 21% in those without a history of cancer.^39^ It is also in agreement with other research into older adults, in a study of over 9000 women with cancer.^40^ In that study, self‐reported physical function was measured by the RAND Short Form 36 scale in women who had a mean age at diagnosis of 73.^40^ Notably in that study, it was identified that from immediately after diagnosis to years later, survivors continue to have lower physical function than cancer‐free controls.^40^ The AYA population would certainly benefit from more longitudinal research comparing their outcomes with those of their cancer‐free peers to identify optimal periods with which to introduce strategies to ameliorate.

Other aspects to consider include the impact on physical function of the type of cancer experienced by AYAs, and the subsequent treatment undergone, as some may lead to specific deficits in physical function. For example, in children and young adults with posterior fossa medulloblastomas, 61% still had weaknesses in locomotion following treatment even 5 years on.^41^ When tested over 5 years on childhood cancer survivors of CNS and bone and soft tissue sarcomas scored the lowest on strength testing of all the cancer types included.^42^ Additionally, in patients with sarcomas, functional status may be better following limb‐saving surgery as opposed to ablative therapy.^43^

A recent systematic review and meta‐analysis identified that physical function could predict mortality in adult patients with cancer.^44^ That study, however, focused on physical function measured anthropometrically as opposed to patient reported, and was not focused on the AYA population—instead, it comprised of mostly older adults.^44^ It emphasises the importance of further research in this field, and it would be interesting to examine the link between patient‐perceived physical function and mortality.

Further research should look at factors associated with worse physical function outcomes in AYA cancer patients and survivors, and what can be done to improve outcomes. For example, some research in older adults has examined effects of a 12‐week physical exercise program. While it did not yield a statistically significant improvement in subjective physical function using PROMs, the effect among the AYA population is unknown.^45^ A systematic review on the effect of exercise on physical function in colorectal cancer patients undergoing chemotherapy also found that there was no clear evidence to suggest that the program improved self‐reported physical function.^46^ Future research could also analyse more closely the questionnaires by individual items, considering this review focused on the overall measurement result.

### 4.1 Limitations

This review has some limitations. The physical function outcome does not encompass physical activity, which would also be a valuable outcome to explore and which may have some cross over with physical function.

Second, the instruments included are very different, so it can be challenging to consider the results altogether. For example, the EORTC‐QLQ‐C30 and FACT‐G are cancer‐specific questionnaires. Meanwhile, the SF‐36 and SF‐12 are not cancer‐specific and instead are broader health surveys that can be used in many chronic conditions. In addition to this, the instruments are validated in adult populations but not necessarily in AYA populations^47^—therefore, there may be some under‐ or overestimation of the PROMs.

Because our search included only full‐text English language articles, some potentially valuable studies published in another language may have been excluded.

A handful of studies used population norm values as a control group, and this is a further limitation. Due to the difficulty making gender and age comparisons, and the potential for the group of interest to have unique distributions, it can be challenging to compare the SF‐36 responses of a group with an illness to normative data from a population.^48^

## 5 CONCLUSION

It is clear from this systematic review and meta‐analysis that AYAs post a cancer diagnosis have worse physical function outcomes compared to their cancer‐free peers. Data are sparse regarding whether there are other risk factors or associations with physical function outside of the cancer diagnosis and subsequent treatment. Further research would be beneficial to identify interventions to improve physical function in this demographic.

## AUTHOR CONTRIBUTIONS

**Sarah Tanner:** Data curation (lead); formal analysis (lead); methodology (equal); visualization (lead); writing – original draft (lead); writing – review and editing (equal). **Teyl Engstrom:** Data curation (supporting); formal analysis (supporting); methodology (equal); project administration (lead); visualization (supporting); writing – original draft (supporting); writing – review and editing (equal). **Cheryl Forbes:** Data curation (supporting); formal analysis (supporting); writing – review and editing (equal). **Dhaval Patel:** Formal analysis (supporting); writing – review and editing (equal). **Wen Ray Lee:** Formal analysis (supporting); writing – review and editing (equal). **Rick Walker:** Validation (equal); writing – review and editing (equal). **Natalie K. Bradford:** Validation (equal); writing – review and editing (equal). **Jason D. Pole:** Conceptualization (lead); supervision (lead); validation (equal); writing – review and editing (equal).

## FUNDING INFORMATION

The authors received no specific funding for this work.

## CONFLICT OF INTEREST STATEMENT

The authors declare no potential conflicts of interest.

## ETHICS STATEMENT

Not applicable.

## Data Availability

Data sharing not applicable as no new data were generated, or the article describes entirely theoretical research.
